# The longitudinal associations between nature exposure and non-suicidal self-injury among Chinese early adolescents: a moderated mediation model

**DOI:** 10.3389/fpsyt.2026.1948760

**Published:** 2026-10-01

**Authors:** Baoyuan Zhu, Rongyao Li, Jiawen Zhao, Xiuchao Wei

**Affiliations:** 1 School of Landscape Architecture, Beijing Forestry University, Beijing, China; 2 Department of Social Sciences, Hebei Sport University, Shijiazhuang, China; 3 School of Humanities and Social Sciences, Beijing Forestry University, Beijing, China; 4 College of Education Science, Bohai University, Jinzhou, China

**Keywords:** gender difference, mindfulness, moderated mediation, nature exposure, non-suicidal self-injury

## Abstract

**Background:**

Non-suicidal self-injury (NSSI) is a major public health concern during adolescence, yet relatively little is known about whether daily exposure to natural environments is prospectively associated with adolescents’ self-reported NSSI frequency. This study examined the longitudinal association between nature exposure and NSSI among Chinese early adolescents, and further tested whether mindfulness mediated this association and whether these pathways varied by gender.

**Methods:**

A three-wave longitudinal design with 6-month intervals was used. A total of 1,854 adolescents participated at T1, and the final analytic sample consisted of 1,048 adolescents who completed all three waves and were successfully matched across waves. Nature exposure was assessed at Time 1, mindfulness at Time 2, and NSSI at Time 1 and Time 3. PROCESS Model 8 was used to test the mediating role of mindfulness and the moderating role of gender, controlling for baseline NSSI, age, parental monthly income, and parental educational level.

**Results:**

T1 nature exposure positively predicted T2 mindfulness (*B* = 0.25, *p* < 0.001), and T2 mindfulness negatively predicted T3 NSSI scores (*B* = -0.03, *p* = 0.008). The direct effect of T1 nature exposure on T3 NSSI was not significant after controlling for covariates (*B* = 0.000, *p* = 0.933). The conditional indirect effect through T2 mindfulness was significant among girls (*B* = -0.0083, 95% Bootstrap CI [-0.015, -0.002]), but not among boys (*B* = -0.0030, 95% Bootstrap CI [-0.008, 0.000]). The index of moderated mediation was significant (Index = 0.0053, 95% Bootstrap CI [0.000, 0.01]).

**Conclusion:**

These findings suggest that T1 nature exposure was prospectively associated with lower subsequent NSSI scores through a mindfulness-related indirect pathway, and that this indirect pathway differed by gender. Given the observational design and the small magnitude of the indirect effects, the findings should be interpreted as prospective associations rather than evidence that nature exposure prevents NSSI or reduces clinical risk.

## Introduction

Nonsuicidal self-injury (NSSI) refers to the direct, deliberate, self-inflicted, and culturally or socially unacceptable destruction of body tissue in the absence of suicidal intent (1). Common forms of NSSI include cutting, scratching, burning, hitting, scraping, and carving the skin (2). Although NSSI is distinct from suicidal behavior, it is associated with substantial psychological distress and increased risk of later suicidal thoughts and behaviors (3, 4). NSSI is especially salient during adolescence. A recent global meta-analysis estimated a pooled lifetime prevalence of 17.7% among adolescents aged 10–19 years (5). NSSI typically emerges between ages 13 and 17, with risk increasing after age 12 and peaking between ages 12 and 16 (2, 6). In China, a recent scoping review estimated the lifetime prevalence of NSSI to be 24.7% among youth (7). Given the high prevalence and potential consequences of NSSI, identifying factors associated with adolescents’ NSSI and clarifying their psychological mechanisms remain important.

Research on adolescent NSSI has mainly focused on individual and interpersonal risk factors, including depression, anxiety, shame, body dissatisfaction, childhood maltreatment, and peer bullying (6, 8). By contrast, less attention has been paid to environmental resources that may be associated with adolescents’ psychological adjustment and self-reported NSSI frequency. Nature exposure has increasingly been recognized as one such environmental resource because contact with natural environments has been linked to stress reduction, improved affective states, restored attentional resources, and better mental health (9–11). Therefore, examining nature exposure may provide a useful environmental perspective for understanding adolescents’ self-reported NSSI frequency.

To organize these theoretical perspectives, the present study adopted an environmental resource–psychological resource–behavioral outcome framework, in which nature exposure was conceptualized as the environmental resource, mindfulness as the proximal psychological resource, and self-reported NSSI frequency as the behavioral outcome. Within this framework, the biophilia hypothesis and Stress Reduction Theory support the expected association between nature exposure and later NSSI scores, Attention Restoration Theory explains the pathway from nature exposure to mindfulness, and the Experiential Avoidance Model explains the pathway from mindfulness to NSSI. Together, these theories support a longitudinal pathway in which T1 nature exposure is expected to be associated with T2 mindfulness, which in turn is expected to be associated with T3 self-reported NSSI frequency.

### Nature exposure and non-suicidal self-injury

Previous studies have shown that exposure to green space is associated with lower levels of adolescent aggressive behavior (12). Given that aggression and NSSI may share emotion-regulation-related antecedents (13), NSSI may also be related to nature exposure. Recent research on Chinese adolescents has also shown that modifiable behavioral and psychological resources are associated with NSSI, suggesting that adolescent NSSI may be linked not only to risk factors but also to broader resource-related processes (14). The biophilia hypothesis suggests that humans have an innate tendency to connect with nature, and contact with nature may contribute to psychological recovery and adaptation (15, 16). From this perspective, nature exposure may serve as an ecological resource associated with lower subsequent NSSI among adolescents.

Stress Reduction Theory further provides a potential explanation for this association (17). Natural environments may reduce stress arousal, alleviate negative affect, and facilitate emotional recovery. Given that NSSI is closely associated with negative affect and maladaptive emotion regulation (13, 18, 19), nature exposure may be associated with lower subsequent NSSI by reducing psychological distress and supporting emotion regulation. Although no study has directly examined the longitudinal association between nature exposure and NSSI, related evidence suggests a potential protective role of nature. Vegetation indices have been negatively associated with suicide mortality across 183 countries or regions (20), while longitudinal research has shown that T1 nature connectedness negatively predicted suicidal thoughts two months later (21).

However, important gaps remain. Previous studies have primarily focused on objective natural environments or subjective nature connectedness, rather than adolescents’ actual direct contact with outdoor natural environments in daily life. The present study therefore focuses on direct outdoor nature exposure, emphasizing a concrete and potentially modifiable form of daily nature contact. In addition, existing research has mainly examined suicide-related outcomes rather than NSSI. Although NSSI does not involve suicidal intent, it is associated with an increased risk of subsequent suicidal thoughts and behaviors (22). Examining whether nature exposure prospectively predicts adolescents’ subsequent NSSI scores may therefore extend existing research on nature and self-injury-related outcomes and provide a basis for future intervention research.

### The mediating effect of mindfulness

Nature exposure may serve as an important environmental resource for adolescent mental health, yet the psychological mechanisms linking it to NSSI remain insufficiently understood. Compared with nature exposure as a relatively distal ecological factor, mindfulness represents a more proximal psychological process involved in how individuals perceive and respond to internal distress. Mindfulness refers to present-moment awareness of internal and external experiences with an attitude of non-judgment and acceptance (23). This capacity may be particularly relevant to NSSI because it enables adolescents to notice distress without immediately responding in automatic or maladaptive ways.

Attention Restoration Theory provides a potential explanation for the association between nature exposure and mindfulness. Natural environments can restore depleted attentional resources through restorative qualities such as soft fascination and being away (24). By gently directing attention toward the immediate environment while providing temporary distance from daily stressors and demands, nature exposure may facilitate the present-moment awareness characteristic of mindfulness. Consistent with this perspective, nature exposure has been positively associated with mindfulness-related processes (25), and experimental and intervention studies suggest that contact with natural environments can promote mindful engagement and psychological restoration (26, 27). More recently, mindfulness was found to mediate the association between green space exposure and perceived stress (28). However, much of this evidence is cross-sectional or based on short-term interventions, leaving it unclear whether adolescents’ everyday nature exposure predicts subsequent mindfulness over time.

Mindfulness may, in turn, be associated with lower subsequent NSSI. According to the Experiential Avoidance Model, NSSI may be maintained because it provides temporary relief from unwanted emotional experiences (18). Mindfulness may reduce this tendency by helping adolescents acknowledge distressing thoughts and emotions without immediately suppressing, escaping from, or acting on them. Greater awareness and nonjudgment may also create psychological distance between emotional distress and behavioral responses, thereby reducing reliance on NSSI as a maladaptive coping strategy.

Empirical evidence supports this possibility. Adolescents with recent NSSI have been found to report lower mindfulness, and mindfulness partially mediated the association between depressive symptoms and NSSI (29). Longitudinal studies further indicate that mindfulness facets predict lower subsequent NSSI through reduced shame-proneness (13), while baseline mindfulness has been associated with lower subsequent NSSI through emotion regulation strategies (30). Together, these findings suggest that mindfulness may serve as a proximal psychological mechanism linking nature exposure with subsequent NSSI.

Accordingly, the present study proposed that mindfulness would mediate the longitudinal association between nature exposure and NSSI. Specifically, higher nature exposure was expected to predict higher subsequent mindfulness, which in turn would predict lower subsequent NSSI.

### The moderating role of gender

Gender was examined as a moderator because the present study focused on whether adolescents may differ in how they engage with and respond to nature exposure as an external environmental resource. In the proposed framework, gender was expected to be most relevant to pathways involving nature exposure, rather than to the mindfulness–NSSI pathway. Nature exposure is a relatively distal ecological factor whose psychological effects may depend on how adolescents perceive and respond to natural environments. In contrast, the association between mindfulness and NSSI is grounded in the Experiential Avoidance Model, which emphasizes a more general self-regulatory process involving awareness, acceptance, and nonjudgment. Accordingly, the present study examined gender as a moderator of the nature exposure–mindfulness pathway and the direct nature exposure–NSSI pathway, but did not hypothesize moderation of the mindfulness–NSSI pathway.

Previous research suggests that the psychological benefits of nature exposure may vary across individuals, although evidence regarding gender differences remains inconsistent. Reviews have noted that associations between nature contact and mental health may depend on individual and contextual characteristics, including gender and age (9), and studies have reported stronger benefits for males, stronger benefits for females, or no significant gender differences (11). These mixed findings suggest that gender may function as a potential boundary condition for the effects of nature exposure.

Gender may be particularly relevant to the pathway from nature exposure to mindfulness. Adolescence is characterized by heightened emotional sensitivity and social-evaluative concerns, and gender differences in emotional processing and emotion regulation may influence how adolescents derive psychological benefits from restorative environments. Indirect evidence comes from school-based mindfulness research: Kang et al. (31) found that mindfulness training increased positive affect among girls but not boys relative to controls. Although this does not directly demonstrate gender differences in responses to nature exposure, it suggests that mindfulness-related psychological benefits may vary by gender. Thus, the association between nature exposure and subsequent mindfulness may also differ between boys and girls.

Gender may also moderate the direct association between nature exposure and NSSI. Previous studies suggest that both NSSI risk and its underlying psychological pathways may differ by gender. For example, Wang et al. (32) found that the mediating role of self-esteem in the association between childhood maltreatment and NSSI differed between boys and girls. These findings indicate that gender may shape how distal risk or protective factors are translated into NSSI-related outcomes. Given that nature exposure may relate to NSSI through emotional recovery and psychological regulation processes, gender was therefore also examined as a potential moderator of the direct association between nature exposure and subsequent NSSI.

Based on the above evidence, the present study hypothesized that gender would moderate both the longitudinal association between nature exposure and subsequent mindfulness and the direct longitudinal association between nature exposure and subsequent NSSI.

### The present study

Based on previous theory and empirical evidence, the present study examined the longitudinal association between nature exposure and adolescents’ NSSI, as well as the underlying psychological mechanism and gender difference in this association. First, this study explored whether T1 nature exposure was associated with T3 NSSI. Second, we examined the mediating role of T2 mindfulness in the relationship between T1 nature exposure and T3 NSSI. Finally, we tested the moderating role of gender in the relationship between T1 nature exposure and T2 mindfulness, as well as in the direct relationship between T1 nature exposure and T3 NSSI. A schematic representation of the proposed model is shown in **Figure 1**. In this model, nature exposure was conceptualized as the environmental resource, mindfulness as the psychological resource, and self-reported NSSI frequency as the behavioral outcome.

**Figure 1:**
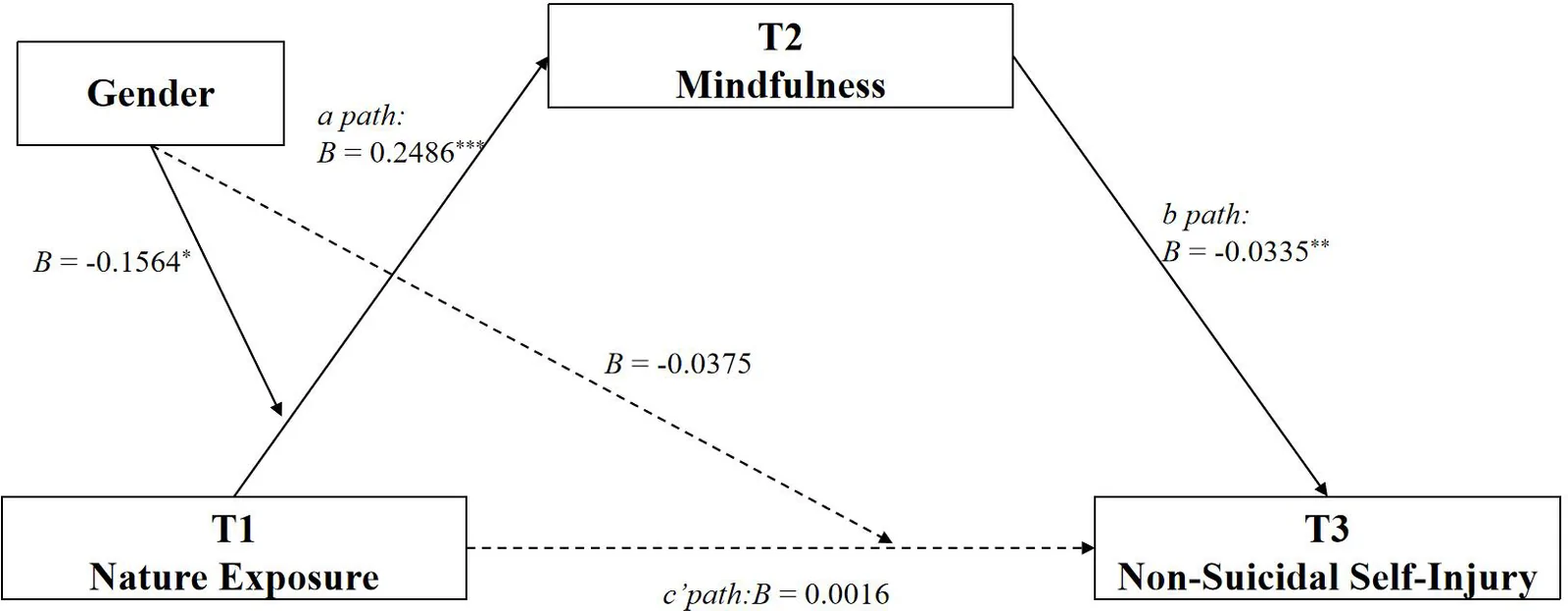
Proposed conceptual model. Moderated mediation model linking T1 nature exposure, T2 mindfulness, and T3 non-suicidal self-injury. Values shown are unstandardized regression coefficients from PROCESS Model 8. Gender was coded as 0 = girls and 1 = boys. Because girls served as the reference group, the coefficients for the paths from T1 nature exposure to T2 mindfulness and from T1 nature exposure to T3 non-suicidal self-injury represent the conditional effects among girls, and the interaction coefficients indicate the difference between boys and girls. Solid lines indicate statistically significant paths, and dashed lines indicate nonsignificant paths. Age, T1 NSSI, fathers’ and mothers’ monthly income, and fathers’ and mothers’ educational level were included as covariates in all analyses but are omitted from the figure for visual simplicity. Full regression results, including covariate estimates, are reported in **Table 4**. ^*^p < 0.05, ^**^p < 0.01, ^***^p < 0.001.

## Methods

### Participants

The study used a three-wave longitudinal design with 6-month intervals between waves. A total of 1,854 students participated at T1 (907 boys and 947 girls), 1, 720 participated at T2 (854 boys and 866 girls), and 1, 517 participated at T3 (740 boys and 777 girls). Gender was self-reported by participants using a binary item and coded as 1 = boys and 0 = girls. For the final analysis, participants were required to have participated in all three waves and to be successfully matched across waves using individual identifiers. A complete-case approach was then applied, and participants with incomplete longitudinal records or invalid responses were excluded. The final analytic sample consisted of 1, 048 adolescents. To examine whether sample attrition was systematic, differences between lost and retained participants were compared on demographic characteristics and NSSI levels at T1. Results showed no significant difference in gender distribution (χ²(1) = 1.08, *p* = 0.298), nor in age (*t*(1839) = 0.27, *p* = 0.787). In contrast, significant differences were found between the two groups regarding paternal monthly income [χ²(4) = 34.17, *p* < 0.001], maternal monthly income [χ²(4) = 35.82, *p* < 0.001], paternal education level [χ²(3) = 12.57, Monte Carlo *p* = 0.004], and maternal education level [χ²(3) = 19.16, Monte Carlo *p* < 0.001], with lost participants generally coming from families with relatively lower socioeconomic resources. Additionally, due to violation of homogeneity of variance, Welch’s independent samples t-test was used to compare T1 NSSI levels between the two groups. The results indicated that lost participants had significantly higher T1 NSSI scores (*M* = 1.17, *SD* = 0.47) than retained participants (*M* = 1.12, *SD* = 0.37), *t*(1452.12) = 2.31, *p* = 0.021. Overall, these findings suggest a certain degree of selective attrition in this study—adolescents from families with limited socioeconomic resources and higher baseline NSSI levels were more likely to drop out during follow-up—but the overall group differences were relatively small.

The final analytic sample was drawn from two schools and 24 classrooms. In the analytic sample, 454 students were from School 1 and 594 students were from School 2. All main regression and conditional process analyses were conducted using this same analytic sample. Participants were between 11 and 16 years old at T1 (*M*_age_ = 13.24, *SD* = 0.86). In terms of gender, 47.6% were boys and 52.4% were girls. Family socioeconomic information was also collected. Regarding fathers’ monthly income, 29.6% reported earning less than 2, 000 RMB, 27.1% reported 2, 000-3, 000 RMB, 17.7% reported 3, 000-4, 000 RMB, 12.8% reported 4, 000-5, 000 RMB, and 12.8% reported 5, 000 RMB or above. For mothers’ monthly income, 47.1% reported earning less than 2, 000 RMB, 24.2% reported 2, 000-3, 000 RMB, 15.2% reported 3, 000-4, 000 RMB, 7.6% reported 4, 000-5, 000 RMB, and 5.8% reported 5, 000 RMB or above. With respect to parental educational level, 80.6% of fathers and 77.2% of mothers had completed junior high school or below, 13.4% of fathers and 14.0% of mothers had completed senior high school or vocational school, 5.7% of fathers and 8.8% of mothers had obtained a college degree or equivalent, and 0.3% of fathers had obtained a graduate degree. Detailed demographic characteristics and participant flow are presented in **Table 1**.

**Table 1:** Demographic characteristics and participant flow.

Characteristic	*n*	%
Participant flow		
T1 participation	1, 854	100.0
T2 participation	1, 720	92.8
T3 participation	1, 517	81.8
Final analytic sample	1, 048	56.5
Gender in final analytic sample		
Boys	499	47.6
Girls	549	52.4
Age at T1		
*M* (*SD*)	13.24 (0.86)	—
Range	11–16	—
Father’s monthly income		
Less than 2, 000 RMB	310	29.6
2, 000–3, 000 RMB	284	27.1
3, 000–4, 000 RMB	186	17.7
4, 000–5, 000 RMB	134	12.8
5, 000 RMB or above	134	12.8
Mother’s monthly income		
Less than 2, 000 RMB	494	47.1
2, 000–3, 000 RMB	254	24.2
3, 000–4, 000 RMB	159	15.2
4, 000–5, 000 RMB	80	7.6
5, 000 RMB or above	61	5.8
Father’s educational level		
Junior high school or below	845	80.6
Senior high school or vocational school	140	13.4
College degree or equivalent	60	5.7
Graduate degree	3	0.3
Mother’s educational level		
Junior high school or below	809	77.2
Senior high school or vocational school	147	14.0
College degree or equivalent	92	8.8
Graduate degree	0	0.0

Percentages for T2 participation, T3 participation, and the final analytic sample were calculated relative to the T1 sample. Percentages for gender, parental income, and parental educational level were calculated based on the final analytic sample (*N* = 1, 048). Income was collected separately for fathers and mothers; total household income was not directly assessed.

### Measures

#### Nature exposure at T1

Nature exposure was assessed using the direct outdoor nature exposure subscale of the Chinese version of the Nature Contact Questionnaire (33, 34). The original NCQ was developed as a 16-item self-report checklist to measure actual nature contact in workplace settings and includes outdoor, indoor, and indirect forms of nature contact (33). In the present study, we used the Chinese adolescent version adopted by Dai et al. (34), which has been applied to Chinese adolescents and distinguishes intentional outdoor nature exposure, intentional indoor nature exposure, and indirect nature exposure. Because the present study focused on adolescents’ direct contact with outdoor natural environments, only the five items assessing direct outdoor nature exposure were used in the main analyses.

The five items assessed adolescents’ direct outdoor contact with natural environments during the past month, such as resting outdoors, eating outdoors, exercising outdoors, engaging in outdoor activities, and appreciating outdoor scenery. Participants rated each item on a 5-point scale ranging from 1 = never to 5 = always. The mean score of the five items was calculated, with higher scores indicating more frequent direct outdoor nature exposure. All items are provided in Appendix A. In the present sample, the subscale showed good internal consistency, with a Cronbach’s α of 0.82. A confirmatory factor analysis was further conducted to examine the dimensionality of the five-item subscale in the current adolescent sample. The one-factor model showed acceptable fit to the data, χ²/df = 7.519, RMSEA = 0.079, GFI = 0.985, CFI = 0.981, IFI = 0.981, TLI = 0.963.

#### Mindfulness at T2

Mindfulness was assessed using the Chinese version of the Child and Adolescent Mindfulness Measure (35). The CAMM was developed to assess mindfulness-related tendencies in children and adolescents and consists of 10 items (36). The Chinese version of the CAMM has been translated, revised, and tested among Chinese adolescents, showing good reliability and validity. In the present study, the CAMM was treated as an overall measure of adolescents’ mindfulness, and the mean score of the 10 items was used in the main analyses.

Participants rated each item on a 5-point scale ranging from 1 = never to 5 = always. Although the original CAMM uses a 0–4 response format, the present study used a 1–5 response format during data collection. This response format is mathematically equivalent to a constant shift of the original scale and does not alter the relative ordering of participants’ responses. All items were reverse scored, and the mean score was calculated, with higher scores indicating higher levels of mindfulness. In the current sample, the CAMM showed good internal consistency, with a Cronbach’s α of 0.92.

#### NSSI at T1 and T3

NSSI was measured using the 10-item version of the Non-Suicidal Self-Injury Scale. The original scale was developed by You et al. (37) and consisted of 12 items. Following previous studies with samples of Chinese adolescents, two items involving chemical reagents were removed, and the remaining 10 items were used to assess adolescents’ NSSI behaviors (38). Items were rated on a 4-point scale (1 = never, 2 = once or twice, 3 = three to five times, and 4 = six or more times). The mean score was calculated, with higher scores indicating a higher frequency of NSSI. The 10 items in the current sample had Cronbach’s α values of 0.92 at T1 and 0.95 at T3.

#### Covariates at T1

T1 NSSI, student age at T1, fathers’ and mothers’ monthly income, and fathers’ and mothers’ educational level were included as planned covariates in all analyses. Fathers’ and mothers’ monthly income and educational level were treated as categorical variables and dummy coded before being entered into the moderated mediation model. For parental monthly income, the lowest income category, less than 2, 000 RMB, was used as the reference group. For parental educational level, junior high school or below was used as the reference group.

### Procedure

The study was conducted using a three-wave longitudinal design, with a 6-month interval between each wave. Participants were recruited from two schools in northern China. Data were collected at Time 1 (T1) in November 2024, Time 2 (T2) in May 2025, and Time 3 (T3) in November 2025. Before data collection, permission was obtained from the participating schools and classroom teachers. Written informed consent forms were distributed to parents or legal guardians, and only students who had returned signed parental consent forms were invited to participate. Before each survey, trained research assistants explained the purpose of the study, the voluntary nature of participation, the principle of confidentiality, and the right to withdraw at any time without penalty. Students who agreed to participate completed the questionnaire; those who did not agree or were absent from school were not included. Written adolescent assent was also obtained before questionnaire completion.

The questionnaires were administered in school computer rooms during regular school hours. Students completed the online questionnaires independently via wjx.cn. Research assistants remained in the room to provide standardized instructions, answer procedural questions, and maintain order, but they did not inspect students’ individual responses. Students’ responses were linked across waves using individual codes. The identifying information used for longitudinal matching was stored separately from questionnaire responses and was accessible only to the research team. Individual responses were not disclosed to classroom teachers or school staff, except when responses indicated recent or severe self-injury and safeguarding follow-up was needed.

Given the sensitive nature of the NSSI items, students were informed that they could skip questions or withdraw from the survey at any time. When responses indicated recent or severe NSSI, the research team followed a safeguarding procedure by informing designated school staff so that further support and follow-up could be provided according to school procedures. Each participant received a notebook as a small gift for participation.

### Data analysis

Data analyses were conducted using Mplus 8.0, SPSS 27.0, and the PROCESS macro developed by Hayes (39). Before testing the moderated mediation model, measurement invariance across gender was examined for the focal multi-item constructs involved in the model. Multi-group confirmatory factor analyses were conducted in Mplus 8.0 using the MLR estimator. Configural, metric, and scalar invariance models were tested sequentially. Model fit was evaluated using χ², *df*, CFI, TLI, RMSEA, and SRMR. Changes in model fit were evaluated using ΔCFI and ΔRMSEA, with |ΔCFI| ≤ 0.010 and |ΔRMSEA| ≤ 0.015 indicating that the more constrained model did not substantially worsen model fit.

After the measurement models were evaluated, subsequent analyses were conducted in SPSS 27.0. Given that all focal constructs were assessed using adolescent self-report measures, common method bias was considered when interpreting the findings. The three-wave longitudinal design provided temporal separation among the focal variables. Harman’s single-factor test was retained only as a limited descriptive diagnostic. PROCESS Model 8 was subsequently used to examine the mediating effect of mindfulness and the moderating role of gender in the relationship between nature exposure and NSSI among adolescents. This model allowed us to test whether gender moderated the first stage of the mediation process, namely the association between T1 nature exposure and T2 mindfulness, as well as the direct effect of T1 nature exposure on T3 NSSI. Prior to interpreting the regression coefficients, multicollinearity diagnostics were conducted by calculating variance inflation factors (VIFs) for all predictors included in the regression models. The bootstrap procedure with 5, 000 resamples was applied to estimate the standard errors and 95% confidence intervals (CIs) of the effects.

## Results

### Measurement invariance analysis

Descriptive statistics and correlations in **Table 2**, and measurement invariance results in **Table 3**. Measurement invariance across gender was examined for the T1 direct outdoor nature exposure subscale. The configural invariance model showed good fit to the data, χ² = 30.24, *df* = 10, CFI = 0.98, TLI = 0.96, RMSEA = 0.06, SRMR = 0.03, indicating that the same one-factor structure was applicable to both boys and girls. The metric invariance model also showed good fit, χ² = 32.69, *df* = 14, CFI = 0.98, TLI = 0.98, RMSEA = 0.05, SRMR = 0.03. Compared with the configural model, the changes in model fit were small, ΔCFI = 0.001 and ΔRMSEA = -0.01, supporting equality of factor loadings across gender. The scalar invariance model further showed acceptable fit, χ² = 44.17, *df* = 18, CFI = 0.98, TLI = 0.97, RMSEA = 0.05, SRMR = 0.04. Compared with the metric model, the changes in model fit remained within the recommended criteria, ΔCFI = -0.01 and ΔRMSEA = 0.003, supporting equality of item intercepts across gender. These results suggest that the T1 direct outdoor nature exposure subscale was measured comparably across boys and girls (**Table 3**).

### Descriptive and correlational analyses

The results of the descriptive and Pearson correlation analyses are presented in **Table 2**. Gender was positively correlated with T1 nature exposure (*r* = 0.10, *p* = 0.001) and negatively correlated with T1 NSSI (*r* = −0.09, *p* = 0.004), but was not significantly associated with T2 mindfulness or T3 NSSI. T1 nature exposure was positively correlated with T2 mindfulness (*r* = 0.18, *p* < 0.001), and negatively correlated with T1 NSSI (*r* = −0.13, *p* < 0.001) and T3 NSSI (*r* = −0.08, *p* = 0.007). T2 mindfulness was negatively correlated with T1 NSSI (*r* = −0.14, *p* < 0.001) and T3 NSSI (*r* = −0.12, *p* < 0.001). Finally, T1 NSSI was positively correlated with T3 NSSI (*r* = 0.25, *p* < 0.001).

**Table 2:** Descriptive statistics and correlations between variables of interest and NSSI.

Variables	1	2	3	4	5
1. Gender	—				
2. T1 Nature exposure	0.10^**^	—			
3. T2 Mindfulness	0.03	0.18^***^	—		
4. T1 NSSI	−0.09^**^	−0.13^***^	−0.14^***^	—	
5. T3 NSSI	−0.06	−0.08^**^	−0.12^***^	0.25^***^	—
Mean	0.48	3.58	3.33	1.12	1.11
*SD*	0.50	0.87	0.95	0.37	0.38

NSSI, non-suicidal self-injury; Gender was dummy coded (0=female, 1=male); T1, Time 1; T2, Time 2; T3, Time 3. ^*^p < 0.05, ^**^p < 0.01, ^***^p < 0.001.

**Table 3:** Measurement invariance across gender for the T1 direct outdoor nature exposure subscale.

Model	χ²	df	χ²/*df*	CFI	TLI	RMSEA	90% CI for RMSEA	SRMR	ΔCFI	ΔRMSEA
Configural invariance	30.242	10	3.02	0.981	0.962	0.062	[0.038, 0.088]	0.027	—	—
Metric invariance	32.689	14	2.33	0.982	0.975	0.050	[0.028, 0.073]	0.029	0.001	-0.012
Scalar invariance	44.174	18	2.45	0.975	0.973	0.053	[0.033, 0.073]	0.037	-0.007	0.003

To examine whether the main findings were robust to the floor-concentrated distribution of NSSI, a supplementary binary logistic regression analysis was conducted using T3 any-NSSI as the outcome variable. Participants were coded as reporting any NSSI if they endorsed at least one of the ten NSSI behaviors above “never.” The model included the same focal predictors and covariates as the main analysis. The overall logistic regression model was significant, χ²(19) = 101.944, *p* < 0.001, with a Nagelkerke R² of 0.152. The Hosmer–Lemeshow test was not significant, χ²(8) = 10.594, *p* = 0.226, suggesting acceptable model fit. Consistent with the main analysis, T2 mindfulness significantly predicted lower odds of T3 any-NSSI (*B* = −0.35, *SE* = 0.10, Wald = 13.90, *p* < 0.001, *OR* = 0.703). T1 nature exposure did not significantly predict T3 any-NSSI (*B* = −0.20, *SE* = 0.131, Wald = 2.40, *p* = 0.12, *OR* = 0.82), and the interaction between T1 nature exposure and gender was also not significant (*B* = −0.10, *SE* = 0.20, Wald = 0.25, *p* = 0.62, *OR* = 0.91). These results suggest that the main conclusions were not solely driven by treating the skewed NSSI mean score as a continuous outcome.

### Distribution of NSSI scores and robustness analysis

The distribution of NSSI scores was examined before model testing. T1 NSSI showed a floor-concentrated and positively skewed distribution, with a mean of 1.12, median of 1.00, *SD* of 0.37, skewness of 4.61, and kurtosis of 24.88. A total of 802 adolescents (76.5%) reported no NSSI at T1, whereas 246 adolescents (23.5%) reported at least one NSSI behavior. T3 NSSI showed a similar floor-concentrated distribution, with a mean of 1.11, median of 1.00, *SD* of 0.38, skewness of 5.04, and kurtosis of 28.71. A total of 860 adolescents (82.1%) reported no NSSI at T3, whereas 188 adolescents (17.9%) reported at least one NSSI behavior. These results indicated that NSSI scores were concentrated at the lower end of the scale.

### Model testing

After entering the data into PROCESS and controlling for covariates, the mediating effect of mindfulness and the moderating effect of gender were tested using Model 8. T1 NSSI, student age at T1, fathers’ and mothers’ monthly income, and fathers’ and mothers’ educational level were included as covariates in all analyses. Fathers’ and mothers’ monthly income and educational level were treated as categorical variables and entered using dummy coding. For parental monthly income, the reference category was less than 2, 000 RMB. For parental educational level, the reference category was junior high school or below. All moderated mediation analyses were conducted using the final analytic sample of 1, 048 adolescents. Missing data were handled using a complete-case approach, and the same analytic sample was used across the regression models. Before interpreting the regression coefficients, multicollinearity diagnostics were conducted for all predictors included in the regression models. The variance inflation factor (VIF) values ranged from 1.025 to 2.118, suggesting that multicollinearity was not a concern. The results are shown in **Table 4**, where T3 NSSI was the outcome variable, T1 nature exposure was the predictor variable, T2 mindfulness was the mediator variable, and gender was the moderator variable. The results indicated that T1 nature exposure significantly and positively predicted T2 mindfulness (*B* = 0.25, *p* <.001, 95% CI [0.16, 0.34]). The interaction between T1 nature exposure and gender also significantly predicted T2 mindfulness (*B* = −0.16, *p* = 0.025, 95% CI [−0.29, −0.02]), whereas the interaction between T1 nature exposure and gender did not significantly predict T3 NSSI (*B* = −0.04, *p* = 0.202, 95% CI [−0.10, 0.02]). T2 mindfulness significantly and negatively predicted T3 NSSI (*B* = −0.03, *p* = 0.008, 95% CI [−0.06, −0.01]). However, the direct effect of T1 nature exposure on T3 NSSI was not significant (*B* = 0.002, *p* = 0.933, 95% CI [−0.04, 0.04]). The interaction term indicated a significant moderating role of gender in the relationship between T1 nature exposure and T2 mindfulness (**Figure 2**). A simple slope test controlling for covariates showed that the effect of T1 nature exposure on T2 mindfulness was greater in girls (*B* = 0.25, *p* < 0.001, 95% CI [0.16, 0.34]) than in boys (*B* = 0.09, *p* = 0.077, 95% CI [−0.01, 0.19]).

**Table 4:** Results of regression analysis testing the role of mindfulness as a mediator, and gender as a moderator, of the longitudinal relationship between nature exposure and NSSI.

Regression models	Variables	*B*	*SE*(HC3)	*t*	*p*	LLCI	ULCI
Model 1: Outcome=T2 mindfulness	T1 nature exposure	0.2486	0.0463	5.3655	<.001	0.1577	0.3395
	gender	-0.0101	0.0594	-0.1708	0.8645	-0.1267	0.1064
	T1 nature exposure × Gender	-0.1564	0.0696	-2.2479	0.0248	-0.2929	-0.0199
	T1_NSSI	-0.3054	0.0970	-3.1474	0.0017	-0.4958	-0.1150
	Age	-0.0752	0.0355	-2.1172	0.0345	-0.1448	-0.0055
Father’s monthly income	2000–2999 RMB	-0.0330	0.0871	-0.3794	0.7045	-0.2040	0.1379
	3000–3999 RMB	0.0976	0.1006	0.9702	0.3322	-0.0998	0.2951
	4000–4999 RMB	0.0082	0.1162	0.0704	0.9439	-0.2199	0.2363
	≥5000 RMB	-0.1933	0.1237	-1.5636	0.1182	-0.4360	0.0493
Mother’s monthly income	2000–2999 RMB	0.0337	0.0840	0.4010	0.6885	-0.1311	0.1985
	3000–3999 RMB	-0.1364	0.1076	-1.2684	0.2050	-0.3475	0.0746
	4000–4999 RMB	0.0480	0.1258	0.3817	0.7028	-0.1989	0.2950
	≥5000 RMB	0.5193	0.1480	3.5082	0.0005	0.2288	0.8097
Father’s educational level	Senior high school/vocational school	-0.0296	0.0921	-0.3218	0.7477	-0.2103	0.1511
	College degree or equivalent	0.0638	0.1583	0.4030	0.6870	-0.2468	0.3743
	Graduate degree	0.3396	0.3266	1.0397	0.2987	-0.3013	0.9805
Mother’s educational level	Senior high school/vocational school	0.1805	0.0956	1.8876	0.0594	-0.0071	0.3682
	College degree or equivalent	0.2075	0.1270	1.6334	0.1027	-0.0418	0.4567
Model 2: Outcome=T3 NSSI	T1 nature exposure	0.0016	0.0194	0.0839	0.9332	-0.0364	0.0397
	T2 mindfulness	-0.0335	0.0126	-2.6478	0.0082	-0.0582	-0.0087
	gender	-0.0165	0.0242	-0.6826	0.4950	-0.0641	0.0310
	T1 nature exposure × Gender	-0.0375	0.0294	-1.2775	0.2017	-0.0951	0.0201
	T1_NSSI	0.2527	0.0618	4.0912	<.001	0.1315	0.3739
	Age	-0.0063	0.0146	-0.4333	0.6649	-0.0349	0.0223
Father’s monthly income	2000–2999 RMB	0.0026	0.0331	0.0780	0.9379	-0.0624	0.0675
	3000–3999 RMB	-0.0474	0.0415	-1.1433	0.2532	-0.1289	0.0340
	4000–4999 RMB	-0.1049	0.0432	-2.4278	0.0154	-0.1897	-0.0201
	≥5000 RMB	-0.0643	0.0469	-1.3721	0.1703	-0.1563	0.0277
Mother’s monthly income	2000–2999 RMB	0.0320	0.0295	1.0847	0.2783	-0.0259	0.0898
	3000–3999 RMB	0.0883	0.0529	1.6704	0.0951	-0.0154	0.1921
	4000–4999 RMB	0.0664	0.0430	1.5447	0.1227	-0.0179	0.1507
	≥5000 RMB	0.0203	0.0336	0.6048	0.5455	-0.0456	0.0861
Father’s educational level	Senior high school/vocational school	0.0434	0.0501	0.8661	0.3866	-0.0549	0.1417
	College degree or equivalent	-0.0668	0.0429	-1.5561	0.1200	-0.1511	0.0174
	Graduate degree	0.9692	1.2796	0.7575	0.4489	-1.5417	3.4802
Mother’s educational level	Senior high school/vocational school	0.0344	0.0449	0.7650	0.4445	-0.0538	0.1225
	College degree or equivalent	0.0005	0.0552	0.0098	0.9922	-0.1077	0.1088

For fathers’ and mothers’ monthly income, the reference category was less than 2, 000 RMB. For fathers’ and mothers’ educational level, the reference category was junior high school or below. Each income or education coefficient represents the difference between that category and the corresponding reference category. Model 1 predicted T2 mindfulness, and Model 2 predicted T3 NSSI. ^*^p <.05, ^**^p <.01, ^***^p <.001.

**Figure 2:**
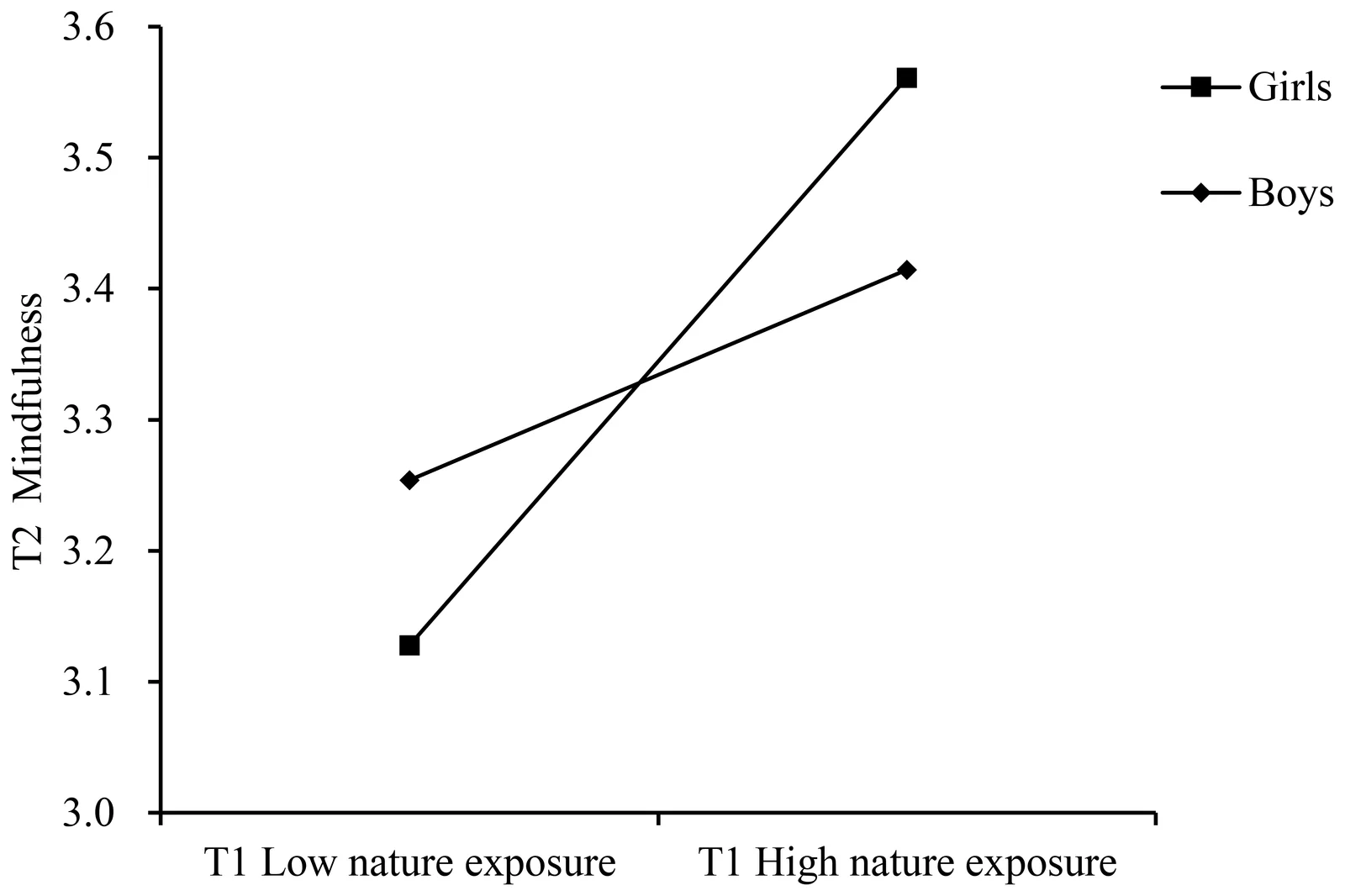
Moderating effect of gender on the relationship between nature exposure and mindfulness.

The conditional indirect effect of T1 nature exposure on T3 NSSI through T2 mindfulness was examined using bootstrap procedures with 5, 000 resamples. The indirect effect was significant among girls (*B* = -0.01, 95% Bootstrap CI [-0.02, -0.002]), whereas the indirect effect among boys was not significant (*B* = -0.0030, 95% Bootstrap CI [-0.01, 0.000]). The index of moderated mediation was significant (Index = 0.01, 95% Bootstrap CI [0.000, 0.01]), suggesting that the indirect association between nature exposure and NSSI through mindfulness differed by gender.

## Discussion

### Nature exposure and non-suicidal self-injury

The present study first examined the longitudinal association between nature exposure and NSSI among adolescents. Nature exposure at T1 was negatively correlated with NSSI at T3, indicating that adolescents with greater nature exposure subsequently reported lower NSSI. This finding is broadly consistent with evidence linking nature exposure to better mental health, stress recovery, and affective functioning (10). Given that NSSI is often used to cope with intense negative emotions or internal distress, nature exposure may be relevant to later NSSI through its associations with stress reduction and emotional recovery.

However, after controlling for T1 NSSI, T2 mindfulness, gender, age, and family socioeconomic status, the direct association between T1 nature exposure and T3 NSSI was not significant. This suggests that nature exposure may be associated with NSSI primarily through more proximal psychological processes rather than through a direct pathway. Consistent with this interpretation, Markevych et al. (40) proposed that the health benefits of natural environments may operate through multiple pathways, including psychological restoration and the development of individual capacities. Nature exposure may therefore function as a relatively distal ecological resource whose association with NSSI depends on psychological mechanisms such as mindfulness and emotion regulation.

The stability of NSSI may also help explain the nonsignificant direct effect. T1 NSSI significantly predicted T3 NSSI, consistent with longitudinal evidence showing that prior NSSI is an important predictor of future NSSI (41). Once baseline NSSI was controlled, nature exposure may therefore have had limited additional predictive value. Nevertheless, the mediation analysis revealed a significant indirect pathway: T1 nature exposure positively predicted T2 mindfulness, which in turn negatively predicted T3 NSSI. This pattern suggests that nature exposure may be prospectively associated with lower NSSI through mindfulness-related psychological processes. Previous research has similarly shown that mindful observation may strengthen the mental health benefits of nature contact (11), while mindfulness has been linked to lower subsequent NSSI through more adaptive emotion-regulation strategies (30). However, because baseline mindfulness was not assessed, the association between T1 nature exposure and T2 mindfulness should not be interpreted as evidence that nature exposure increased mindfulness over time.

The magnitude of these associations should also be interpreted cautiously. The zero-order association between T1 nature exposure and T3 NSSI was small, and the adjusted direct effect was close to zero. Although the conditional indirect effect through mindfulness was statistically significant among girls, its absolute magnitude was also small. Thus, the findings indicate a modest prospective association and provide preliminary evidence for a mindfulness-related pathway that warrants further experimental and intervention research.

### The mediating effect of mindfulness

The present study identified a significant conditional indirect association between nature exposure and adolescent NSSI through mindfulness. Specifically, T1 nature exposure positively predicted T2 mindfulness, which in turn negatively predicted T3 NSSI. This finding suggests that mindfulness may serve as a proximal psychological process linking nature exposure with subsequent NSSI.

The association between nature exposure and subsequent mindfulness may be explained by the restorative qualities of natural environments. According to Attention Restoration Theory, exposure to nature can facilitate recovery from directed-attention fatigue by providing a relatively undemanding context in which attentional resources can be restored (24). This restorative process is consistent with the present-moment awareness emphasized in mindfulness. By temporarily disengaging attention from everyday demands and stress-related cues, natural environments may facilitate greater awareness of immediate internal and external experiences. Consistent with this interpretation, previous research has linked nature exposure to mindfulness-related processes (25). The present findings extend this literature by showing that greater nature exposure was associated with higher subsequent mindfulness. However, because baseline mindfulness was not assessed, this association should not be interpreted as evidence of an increase in mindfulness over time.

Mindfulness, in turn, negatively predicted subsequent NSSI. According to the Experiential Avoidance Model, NSSI may be maintained because it provides short-term relief from aversive emotional experiences (18). Mindfulness may reduce reliance on such avoidance-based responses by helping adolescents recognize difficult emotions and self-injurious urges with greater awareness, acceptance, and less judgment. In this way, mindfulness may be associated with a greater capacity to tolerate distress without immediately responding through self-injury.

This interpretation is consistent with recent longitudinal research among adolescents. Yuan et al. (30) found that baseline mindfulness predicted lower subsequent NSSI through emotion-regulation strategies, including cognitive reappraisal and expressive suppression. Zhang et al. (13) similarly showed that several mindfulness dimensions predicted lower subsequent NSSI through reduced shame proneness. Together, these findings suggest that mindfulness may be associated with lower subsequent NSSI through multiple processes, including more adaptive emotion regulation, reduced negative self-evaluation, and greater tolerance of distress.

### The moderating role of gender

The present findings provided partial support for the moderating role of gender. Gender significantly moderated the association between T1 nature exposure and T2 mindfulness, with the positive longitudinal association being more evident among girls than boys in the present sample. However, gender did not significantly moderate the direct association between T1 nature exposure and T3 NSSI. This pattern suggests that gender may be more relevant to the pathway linking nature exposure with later mindfulness than to the direct pathway linking nature exposure with NSSI.

The stronger nature exposure–mindfulness association among girls should be interpreted cautiously. The interaction effect was modest, and no directional gender hypothesis was specified *a priori*. One possible explanation is that gender-related differences in emotional processing, stress responses, or socialization may influence how adolescents engage with restorative environments and mindfulness-related experiences. Girls have been found to report greater emotional intensity and expression of internalizing emotions than boys (42), and school-based mindfulness research has shown greater improvements in positive affect among girls than boys following mindfulness training (31). Although these findings provide indirect support for gender-related differences in mindfulness-related benefits, they do not establish that girls are inherently more responsive to nature exposure. The present result should therefore be regarded as a preliminary, sample-specific pattern.

By contrast, gender did not significantly moderate the direct association between nature exposure and NSSI. Previous research suggests that the mental health benefits of nature exposure may vary across gender and age groups (9), but these studies have generally focused on broader outcomes such as psychological distress, affect, or well-being. NSSI represents a more specific behavioral outcome influenced by multiple proximal factors, including prior self-injury, emotion-regulation difficulties, negative self-evaluation, and interpersonal stress. Thus, gender differences in responses to nature exposure may be less readily expressed in the direct association between a relatively distal environmental resource and subsequent NSSI.

It is also important to distinguish gender differences in NSSI risk from the moderating role of gender in specific predictive pathways. Previous research has shown that females are more likely than males to report NSSI (43), and that some psychological pathways to NSSI may differ by gender (32). However, such findings do not necessarily imply that gender moderates the direct association between nature exposure and NSSI. In the present study, gender differences appeared more evident in the nature exposure–mindfulness pathway. This interpretation remains preliminary and should be tested in future research incorporating more detailed assessments of gender-related emotional, social, and contextual processes.

### Limitations and future research

Several limitations should be acknowledged. First, although the three-wave longitudinal design established temporal ordering among T1 nature exposure, T2 mindfulness, and T3 NSSI, the study remained observational and cannot support definitive causal conclusions. Because mindfulness was assessed only at T2, the present study could not determine whether nature exposure predicted changes in mindfulness over time or reflected pre-existing individual differences in mindfulness. Future research should assess all focal variables repeatedly across multiple waves and use cross-lagged panel models, random-intercept cross-lagged panel models, or other dynamic analytic approaches, such as cross-lagged network analysis, to clarify directional and within-person processes (44). In addition, the present study examined self-reported NSSI frequency scores rather than NSSI onset, recurrence, cessation, or clinical risk status. Therefore, the findings should be interpreted as prospective associations with reported NSSI scores rather than evidence of NSSI risk reduction or prevention.

Second, the measurement of nature exposure and NSSI could be refined. Nature exposure was assessed by adolescent self-report and did not distinguish among the type, quality, duration, or context of nature contact. Future studies could integrate objective and intensive assessment methods, such as green-space indicators, GPS tracking, ecological momentary assessment, and daily diary methods. NSSI is also a sensitive behavior and may be affected by recall bias, social desirability, and willingness to disclose. Future research could combine self-report measures with clinical interviews or multi-informant reports and distinguish between the occurrence, frequency, and severity of NSSI.

Third, the present study provided limited insight into the specific psychological processes underlying the indirect pathway. Mindfulness was analyzed as a global construct, without differentiating among facets such as present-moment awareness and nonjudgmental acceptance. Future studies could examine distinct mindfulness components and additional mechanisms, including emotion regulation, rumination, self-compassion, shame, and distress tolerance. In addition, gender was assessed as a binary category, which limited the ability to capture broader gender identities and gender-related social experiences. The gender moderation finding should therefore be interpreted cautiously and tested in future research using more nuanced assessments of gender-related processes.

Finally, the sample consisted of early adolescents recruited from two schools in northern China, which may limit generalizability. Participants were also nested within 24 classrooms, but classroom-level clustering was not formally modeled in the primary conditional process analyses. Thus, standard errors may not have fully reflected the nested data structure. Future studies should recruit larger and more diverse samples across regions, school contexts, urban and rural settings, and developmental stages, and should use multilevel models or cluster-robust approaches to account for school-based clustering. Moreover, some selective attrition was observed. Participants lost to follow-up tended to come from families with lower parental education and income and reported slightly higher baseline NSSI than retained participants. These differences were generally small but may limit sample representativeness. Because the specific reasons for attrition were not assessed, the underlying mechanisms remain unclear.

### Implications

The present findings offer a preliminary perspective on the role of nature exposure in adolescent self-reported NSSI frequency. Rather than suggesting that nature exposure directly prevents NSSI, the results indicate that nature exposure may be prospectively associated with lower subsequent NSSI scores through mindfulness-related psychological processes. Future experimental and intervention studies are needed to test whether structured nature-based activities, particularly when combined with mindfulness-related practices, can reduce self-reported NSSI frequency or lower the likelihood of NSSI onset. Any such intervention should be implemented alongside established NSSI assessment procedures, safeguarding protocols, and clinical referral mechanisms.

## Conclusions

This study used a three-wave longitudinal design to examine the association between nature exposure and self-reported NSSI among Chinese early adolescents. T1 nature exposure positively predicted T2 mindfulness, and T2 mindfulness negatively predicted T3 NSSI scores. The direct association between T1 nature exposure and T3 NSSI was not significant after controlling for baseline NSSI and relevant demographic variables. The conditional indirect effect indicated that T1 nature exposure was indirectly associated with lower T3 NSSI scores through T2 mindfulness, and the significant index of moderated mediation suggested that this indirect pathway differed by gender. Given the observational design and the small magnitude of the effects, the findings should be interpreted as modest prospective associations rather than evidence that nature exposure prevents NSSI or reduces clinical risk.

## Data Availability

The datasets generated and/or analyzed during the current study are not publicly available due to ethical and privacy considerations concerning adolescent participants and sensitive NSSI-related data, but de-identified data may be available from the corresponding author upon reasonable request and subject to appropriate ethical and institutional approval.

## APPENDIX

**Appendix A:** Please indicate the number that best represents your actual situation during the past month by marking “O”.

Item	Items	1 = Never	2 = Rarely	3 = Sometimes	4 = Often	5 = Always
1	You have rested outdoors, such as lying in the sun.	1	2	3	4	5
2	You have eaten outdoors, such as dining outside or having street food.	1	2	3	4	5
3	You have exercised outdoors, such as taking a walk after meals.	1	2	3	4	5
4	You have engaged in outdoor activities, such as outdoor work or classes.	1	2	3	4	5
5	You have gone outdoors or to open spaces to appreciate the scenery.	1	2	3	4	5
